# The first Finnish malariologist, Johan Haartman, and the discussion about malaria in 18th century Turku, Finland

**DOI:** 10.1186/1475-2875-10-43

**Published:** 2011-02-15

**Authors:** Lena Hulden

**Affiliations:** 1 Department of Agricultural Sciences, Faculty of Agriculture and Forestry, University of Helsinki, Finland

## Abstract

After the Great Northern War in 1721, Sweden ceased to be an important military power. Instead, the kingdom concentrated on developing science. Swedish research got international fame with names as Carolus Linnaeus, Pehr Wargentin and Anders Celsius. Medical research remained limited and malaria was common especially in the coastal area and along the shores of the big lakes.

Already in the beginning of the 18^th ^century Swedish physicians recommended Peruvian bark as medication and they also emphasized that bleeding or blood-letting a malaria patient was harmful. Although malaria was a common disease in the kingdom, the situation was worst in the SW-part of Finland which consisted of the town of Turku and a large archipelago in the Baltic. The farmers had no opportunity to get modern healthcare until Johan Haartman was appointed district physician in 1754. To improve the situation he wrote a medical handbook intended for both the farmers and for persons of rank.

Haartman's work was first published 1759 and he discussed all the different cures and medications. His aim was to recommend the best ones and warn against the harmful. His first choice was Peruvian bark, but he knew that the farmers could not afford it.

Haartman was appointed professor in medicine at the Royal Academy of Turku in 1765. The malaria situation in Finland grew worse in the 1770's and Haartman analysed the situation. He found the connection between the warm summers and the spring epidemics next year.

In a later thesis, Haartman analysed the late summer/early autumn malaria epidemics in the archipelago. Althouh Haartman did not know the connection between malaria and the vector, he gave astute advice and encouraged the farmers to build their cottages in windy places away from the shallow bays in which the *Anopheles *females hatched. Haartman died in 1788. After his death malaria research in Turku declined. His medical handbook would not be replaced until 1844.

## Background

After the Great Northern War (1700-1721), Sweden lost the major part of its Baltic provinces and Karelia in Eastern Finland. The autocracy was replaced by the power of the estates of the realm. The military budget was decreased. More resources were invested in the development of universities, academies and research. Swedish science, with names as Anders Celsius, Carolus Linnaeus and Pehr Wargentin, made the Swedish academies and the University of Uppsala internationally important. Natural sciences as astronomy, botany, entomology and chemistry became fashionable and the queen herself had a famous collection of insects [1]. For the first time European scholars started to turn to Uppsala for new scientific results.

In spite of the general progress during the 18^th ^century, medical research remained limited and the results were mainly made in surgery. In the beginning of the century quacks and barber-surgeons usually treated the patients. There were no modern public hospitals. The medical resources had, therefore, to be concentrated on the development of a modern health care system and to provide education, not only for physicians but also for surgeons and midwives. Rampant venereal diseases and rinderpest epidemics were acute problems [1]. Smallpox epidemics caused annually a lot of deaths and many of the survivors suffered from blindness. The disease could be fought by inoculations and the first campaigns were organized towards the end of century [2]. Malaria was a common disease and in many parts of the country a major problem. Malaria research therefore became important in spite of the limited resources [3].

Peruvian bark or Cinchona had changed malaria treatment in Europe and was recommended already in 1643 [4]. The recipe became generally available after 1682 [5]. Francesco Torti published his fundamental work on the therapeutic value of Peruvian bark in 1712 [6]. The Swedish physicians usually travelled abroad for their dissertation in the beginning of the 18^th ^century. The sojourn at a foreign university gave them knowledge about the new advances in medicine. Johan Linder was one of the first to propagate for the new drug in Sweden. He began his studies in medicine in the Royal Academy of Turku (today University of Helsinki) and moved to Uppsala in 1703. He later completed his studies in Harderwijk and Leiden in the Netherlands [7]. In 1717, he published his first study of malaria and the use of Peruvian bark [8]. Linder's work was a synthesis of the international research, as far as he knew it, combined with an analysis of the situation in Sweden. He discussed several different theories on the effectiveness of Peruvian bark and finally reached the same conclusion as Sebastian Badi, namely that he did not know [8].

Swedish malaria research was continued by Carolus Linnaeus. He also travelled to Harderwijk where he defended a thesis on the cause of malaria and took his degree in less than two weeks [1]. Linnaeus' contribution to medical research has been considered unfortunate, especially when he tried to explain the cause of diseases [1]. The ultimate cause of malaria was, according to him, particles of clay that had been dissolved in the drinking water [9]. In a later work, Linnaeus analysed malaria in his hometown Uppsala. The town was so pestered by malaria that the parents refused to send their sons to the university there [10].

Both Linder and Linnaeus recommended the use of Peruvian bark although it went against the Galen tradition [5]. The recommendation was followed by most of the Swedish physicians during the 18th century. But the introduction of a new drug went slowly and had little practical effect. It was simply too expensive for the common people. As late as 1856, quinine was still not offered as first choice for malaria in a Finnish medical handbook for farmers by the physician Elias Lönnrot [11].

Galen had prescribed bleeding or blood-letting as a cure for malaria. It was still common during the end of the 19^th ^century in folk medicine used for malaria in South Italy [12]. Even in academic medicine it remained a popular therapy for many diseases in the 18^th ^and partly also in the 19^th ^century [13]. Still the 18^th ^century Swedish physicians broke with Galen also concerning bleeding as malaria therapy. Linder emphasized that bleeding a malaria patient was harmful; it made the patient worse and should at all cost be avoided [8]. Linnaeus was also critical to bleeding a malaria patient. His thesis on the malaria situation in Uppsala ended with the statement: *Vene sectionem nullum effectum salutarem, sed potius noxium, in hoc morbo, ut in intermittermittentibus, praestare vidi *(that to his experience bleeding such a patient was not beneficial but deleterious)[10].

Most of the Swedish research was done in Uppsala and Stockholm. The University of Tartu had become lost in the war. Lund and Turku were small and attracted mostly youngsters from the surrounding provinces. The university in Greifswald had a bad reputation because the students could even get their degree by mail without visiting it [1]. The Royal Academy of Turku had suffered severely and was closed during the war. It could re-open only in 1722 [1]. It had only one chair in medicine and its holder, professor Johan Leche, had few students. He was mainly interested in mineralogical research and in collecting meteorological data [14]. The situation was to change after his death in 1764. The next professor in medicine, Johan Haartman, became the first in Turku to study malaria and discuss different cures and medication.

**Figure 1:**
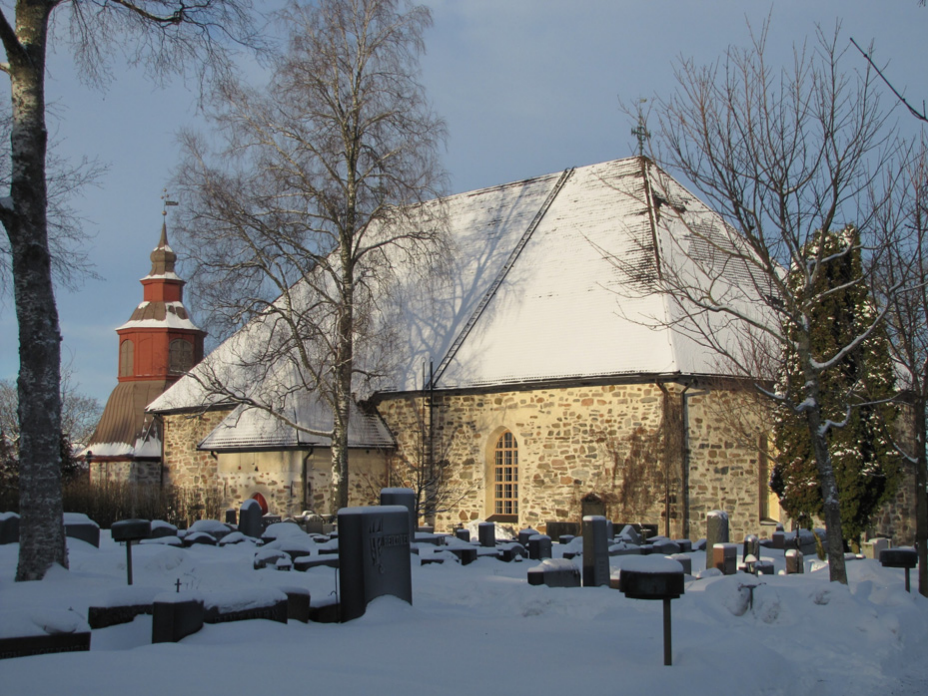
**The church in Paimio, the birthplace of Johan Haartman**. When Johan Haartman was borne his father was professor in theology and the minister in the parish of Paimio, 25 km east from Turku. The vicarage was replaced with a new building in 1806. Haartman's home in Turku, a possible portrait and other mementoes were destroyed in the great fire of the town of Turku in 1827. Only a few letters, the publications and the funds for a chair in medicine, which Haartman donated to the university, remain.

Johan Haartman was borne in 1725 [Figure 1]. His family had been impoverished during the war, so he decided to become a pupil in a pharmacy in Stockholm. After four years he began to attend lectures at the university in Uppsala. His poverty made it impossible for him to buy a pharmacy of his own. Instead he started to study medicine at Uppsala. His main teacher was Nils Rosén von Rosenstein, the author of the first modern paediatric textbook, *The Diseases of Children and Their Remedies *[1].

In 1754 Haartman was appointed district physician in Turku. The number of graduated physicians in Finland was small. Beside the professor in medicine in the Academy there were only four district physicians [15]. During the next years Haartman travelled frequently at his own expense in his district and tried to offer modern health care to the inhabitants [7]. The situation was far from satisfactory and he explained that the Finns had a higher mortality than the people in Sweden. It was due to the scarcely populated country with large areas of wetlands that caused malarial fevers. The sick had far to travel to reach a minister or a parish clerk. They often even lacked close neighbours. Their only retreat was the Finnish sauna, which in the case of malaria did more harm than good [15].

### The malaria situation in Turku area and Haartman's view on medication

Malaria was a common disease in Finland during the 18^th ^century. It declined slowly and the last indigenous case was diagnosed in 1954 [16]. The town of Turku and the SW-archipelago were considered extremely malarious [9]. The disease was often discussed in the local newspaper. The physician Johan Gabriel Bergman wrote several articles about the situation. The climate in Turku favoured malarial fevers, especially among the poor. They had to live in humid cottages and stuffy rooms. Their clothing was inadequate and of bad quality. Because of their small wardrobe they usually had no opportunity to change wet clothes to dry [17].

Malaria had a negative impact on the local economy in the parishes. The minister Erik Lencqvist described the situation in the archipelago. He had personal experience because he had been working in 1750-1752 in the parish of Taivassalo 50 km from Turku. The *autumn malaria *epidemics broke out at the end of summer or at the end of the dog days. The sick suffered for five to six weeks. The sickness became an economic disaster for the farmers. On some farms everyone was ill and in several villages there were nobody fit to work. It became almost impossible to get the harvest gathered in and threshed. The milking of the cows became difficult [18]. On the Aland Islands the situation were even worse than in Turku. The district physician there, Fredric Wilhelm Radloff reported that one person out of forty had died of malaria in 1749-1773 [19].

**Figure 2:**
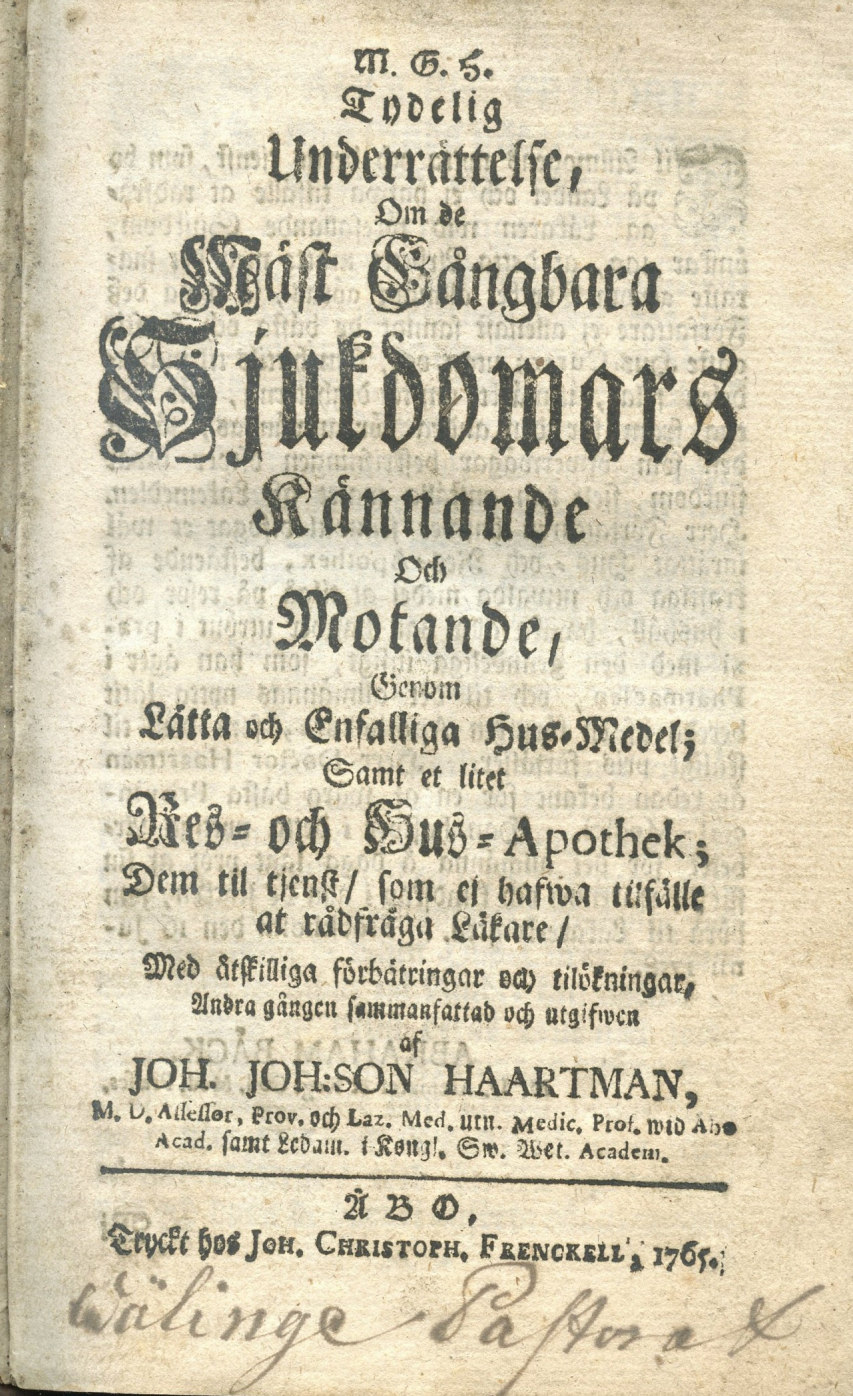
**Johan Haartman's medical handbook**. *Clear advice about recognising and curing the most common diseases with plain and simple medication, and a small apothecary for the homes and for travel; for those that are unable to ask a physician *gave advice on the treatment of all the common diseases. Haartman had a sharp intellect and in most cases he gave an astute assessment of the cures. Sometimes the style was ironical, as when he pondered about the usefulness of placing a snake skin on the stomach of a woman to strengthen her labour pains. He stated that the only possible benefit from that cure would be that the woman might become astonished and for that reason go into labour.

The need for health care was enormous and Haartman could not personally attend all the patients. Instead he decided to write a handbook about the most efficient cures for the common diseases. Because of his former work in the pharmacy and his early studies in pharmacology he was well acquainted with the different cures and available drugs. His work was typical for his time. It showed a confidence in man and his opportunities. Haartman wanted to make a difference and addressed both the ignorant farmers and persons of rank. The latter were supposed to realize that a good health was precious and good economy [20]. The book *Clear advice about recognizing and curing the most common diseases with plain and simple medication, and a small apothecary for the homes and for travel; for those that are unable to ask a physician *was published in 1759 with an improved edition in 1765. The diseases were analysed in an alphabetical order. Haartman not only gave advice about the most efficient cures but he also analysed the commonly used drugs and evaluated the effectiveness of each cure. The book was written in Swedish but the name of the diseases was also added in Finnish [Figure 2].

Haartman tried to make international malaria research understandable for the common people. He explained the differences between Quotidian, Tertian and Quartan malaria and his first recommendation was the use of the excellent and wonderful Peruvian bark. It did not only cure intermittent fevers but it would also provide the best protection against the damage that dangerous cures could cause [20]. Haartman knew the household remedies and which drugs were available. He wrote that the tradition was different in the separate parts of the kingdom and that the use of the medical herbs varied. To publish all available knowledge about the cures would therefore benefit the whole country, an idea that was typical for his practical compatriots of that time [20].

Malaria or the intermittent fevers were considered easy to identify and very common with the typical alternation between periods of cold, heat and sweating. Even though they usually were mild they still killed a lot of people and caused many sick days [20]. To avoid the disease it was effective to use lovage (*Levisticum officinale*), sweet flag (*Acorus calamus*), angelica (probably *Angelica sylvestris*) or clove (*Allium sativum*) as preventive medicine each morning during autumn and spring [20]. The main cure was, however, Peruvian bark. Those who had malaria for the first time could seldom be helped without it. It was also important to repeat the cure after a while [20]. Peruvian bark prevented the damage that untreated malaria could cause [20]. Unfortunately Peruvian bark was unaffordable by most patients and had to be bought in Stockholm. The first Finnish mill for grinding Peruvian bark was established in 1813 in Turku, but the production stopped after a few years [21].

The common people had to use cheaper drugs than Peruvian bark. To cure the spring epidemics of malaria Haartman, therefore, prescribed alternative cures that he had found effective in his practice. The first to be suggested was: 3.3 or 6.6 g salmiac (ammonium chloride) melted in 32.7 or 65.4 cl whey of "beer cheese" [separately heated milk and beer that then were mixed], which should be drunk two hours before the shaking started, afterwards the sweating could begin. Powdered cuttlefish shell could be used in the same way. Useful plants against malaria were: Hart's tongue fern (*Asplenium scolopendrium*), caraway (*Carum carvi*) curled mint (*Mentha spicata *var. *crispa*), garden angelica (*Angelica archangelica*), lovage (*Levisticum officinale*), parsnip (*Pastinaca sativa*), german camomile (*Matricaria recutita*), milk parsley (*Peucedanum palustre *), wood avens (*Geum urbanum*), water avens (*Geum rivale*), shepherd's-purse (*Capsella bursa-pastoris*), St. Benedict's thistle (*Cnicus benedictus*), yarrow (*Achillea millefolium*), wormwood (*Artemisia absinthium*), mugwort (*Artemisia vulgaris*) and blackcurrant (*Ribes nigrum*). The farmers commonly used goldmoss stonecrop (*Sedum acre*), but northern firmoss (*Huperzia selago*) or stiff clubmoss (*Lycopodium annotinum*) could be alternatives. Elecampane (*Inula helenium*), the bark of rowan, birch or ash could also be useful. Other common and effective ingredients were alcohol, sulphur, gall from bear, salmon and pike [20].

Haartman tried to mention all the different cures and available drugs. He warned strictly against the use of arsenic. Zinc vitriol (ZnSO_4_) should also only be used with the greatest care. The most foolhardy without any respect for human life used a mixture of copper(II) sulphate (CuSO_4_), copper(II) acetate Cu(C_2_H_3_O_2_)_2_, nux vomica and 8-10 berries of mezereon (*Daphne mezereum*).

Other household remedies were also mentioned, but Haartman was not very convinced of their usefulness although he agreed that they sometimes could work. Some people used different spices mixed in aquavit or vine. Others used alum mixed with vinegar, caraway, pepper, alcohol or vine. Another mixture was aquavit with pepper, tobacco, gunpowder and gall of salmon. Haartman was even more sceptic against the several coprophagic cures that were mentioned but he thought that those who digested a spoonful of faeces of dog, cat or hen would at least not be harmed. Only few had been cured by eating snake meat or spider web with pepper in a fig or plum. It was then safer to mix mustard with caraway. An ancient method was to mix spider web with mustard or garlic and put the mixture on the arteries. There were also other recipes of mixtures that could be used with plasters [20].

Malaria could be avoided with a strict diet. Haartman especially recommended juice made of rye sprouts, tea of sage or juniper water with wormwood or mugwort. Butter on bread should be replaced by a mash of juniper berries. Fresh herring should also be avoided and it was best not to eat white cabbage, sauerkraut, hard-boiled eggs or cheese [20].

In some aspects *Clear advice about recognizing and curing the most common diseases with plain and simple medication *still resembled an old medieval herbal with a mishmash of household remedies. That it represented a new modern approach to medicine becomes obvious only when it is compared to contemporary prescriptions. There is a handwritten manuscript from 1760 by the minister Johan Wegelius, who was working in Oulu and Finnish Lapland. He took a deep interest in the health care and nursed his sick parishers [22]. He did not use Peruvian bark and preferred traditional cures and drugs. Only a few of his prescriptions can be found in Haartman's work. Contrary to Haartman, Wegelius thought highly of the coprophagic cures and recommended them. Before a fit of malaria, it was useful to take a mixture of pounded dog faeces with mace, saffron and alum. Dog faeces could be exchanged into human faeces during winter. He also recommended the use of mouse droppings. Some people were also helped by mixing their own urine with milk and letting a dog drink the mixture. The most exotic cure for malaria was to smear the patient's body completely with seal blubber [23].

Compared to Wegelius' manuscript, *Clear advice about recognising and curing the most common diseases with plain and simple cures *represents a scientific approach. Haartman analyses the remedies, discusses their properties and possible usefulness. Although he put forward Peruvian bark as the first and best alternative he was aware of the poverty and that people had to get cheaper options. Haartman also reached his goal in giving the people better advice on health care. He decided to donate 1,200 copies of the second edition to all the parishes in the country [7]. It then became known in all parts of the kingdom and got a tremendous impact.

### The malaria research in Turku

Haartman was appointed to professor in medicine in Turku in 1765 and he would diligently work for the academy until his death in 1788. The malaria situation in Finland grew worse in the 1770s' with many severe epidemics in the area near Turku. Haartman then returned to malaria research and analysed the situation. As most international physicians he thought that the ultimate reason for the epidemics was the quality of the air. The air in some areas was pestered with harmful effluvium, which affected sensitive people. This was especially the case in Finnish coastal areas where fogs and mists were common [24].

Haartman knew well the international malaria research and most of his results are mainly of historical interest. Still he also applied his own empirical evidence in the analyses. There was, according to him a difference between malaria fevers in the autumn and in the spring. The sun affected the life force in spring, which made both nerves and fibres strong. It was then more difficult for the malarial agent to coagulate the juices in the body. Therefore, malaria in autumn needed stronger cures than in the spring. The situation was often made worse by the wrong diet [24].

Haartman tested his hypothesis by comparing the malaria situation with meteorological data. The malaria years were analysed in detail in accordance with temperature, barometric pressure, precipitation, wind and thunder. January 1774 had been unusually cold and June had been the driest month for twenty-six years. The summer 1775 was also very dry and in 1776 there was a general crop failure in rye [24]. Haartman understood intuitively the importance of summer temperature although he obviously not knew the actual context. The malaria epidemics in Finland correlated with a previous warm summer. The *Anopheles *larvae developed in June and July and hatched in the end of summer. After a warm summer there were more available vectors, which then hibernated together with man and caused malaria indoors [25]. Haartman's theory was that a warm summer caused shortage of water in the shallow bays in the archipelago, which made the bodies of the people to warm. Therefore the mists were more harmful than otherwise [24].

Usually the number of malaria cases decreased and almost disappeared during summer [26]. The number started to rise again in August. In a thesis Haartman described it as a special kind of an intermittent fever, which he called *archipelago fever*. It was especially common in those parts of the archipelago, which were characterized by shallow and muddy bays. Also the town of Turku and the adjacent islands were afflicted. It was less common in parishes with rocky shores and deep water. The main cause for the disease, were the inhabitants' small and stuffy cabins. The smell of corpses in the churches had also an impact. This had especially been the case for the large number of fatal cases in the archipelago parishes of Rymättylä and Parainen in 1775, 1776 and 1777 [27]. The theory of the cadaverous smell causing for malaria was controversial. The minister Erik Lenqvist argued against it, although he generally accepted Haartman's other theories [18]. Lencqvist had done a lot of fishing in the archipelago. He agreed that the water were shallow around the malarious islands. But he had personally checked the marin flora and found an abundance of an algae, which he had determined to *Chara culgaris *[stonewort, now *Chara foetida*] [18]. The presence of stonewort had often given him headaches while cleaning the trolling spoon [18].

Johan Gabriel Bergman also commented Haartman's conclusions. He thought that they were correct in most cases. Still Bergman supported Lencqvist's view about the importance of stonewort in the waters. He had also several times noticed the same effect [28]. Bergman emphasized that the number of malaria cases varied annually. The large number of fatal cases was not, according to him, the cause of an increased mortality. Instead it was a resulting effect of the large number of cases in general [28].

Haartman's detailed study showed that his research was not only a compilation of other research. He had empirically investigated the disease in the archipelago and treated a lot of patients. He then compared his clinical notes with both Linneus' study of malaria in Uppsala and Bartholin's descriptions from Denmark. Haartman's original theories of the ultimate cause of the disease are still of more interest than the learned discussion. He pointed out that there were more cases among the farmers, than among persons of rank. The reason was that the farmers worked outdoors and their strength was diminished by perspiration [27].

Haartman never realized the importance of the vector. Still his astute observations showed that he had an uncommon ability of understanding the epidemiology of the disease. Several of the improvements he suggested would have led to a declining number of malaria cases. He stated that nature had given winds as an excellent weapon against the disease [27]. Every farmer should build his cottage on a stony height away from the shallow bays. Especially the sleeping sheds should be placed so that the winds could freely reach them. They should be built higher with more layers of logs [27]. The advice would have lead to a decrease in the number of vectors indoors, which naturally would have had an impact on the severity of the spring epidemics.

Another interesting advice was Haartman's view on sleeping habits. He suggested that the daily rhythm should be changed. Instead of a long night sleep the working schedule should be organized with a couple of hours nap during the day. Then the early morning could also be used for work. It should also be important to cover the body well while sleeping [27]. Since the Finnish malaria vector (*Anopheles messeae*) only bites sleeping people when it is quiet, Haartman's proposal would have shortened the biting period.

Haartman did naturally not know that the disease he described as archipelago fever mainly were relapses of *vivax *malaria. In August there were only uninfected females present of the new generation of *Anopheles *sp. Outdoor temperature was not high enough for sporogony and the heating of the buildings had not yet started [25]. Still his advice worked. Mosquitoes avoid wind and fly farther in calm conditions. They are most abundant near the hatching places, which in the Finnish archipelago were the shallow bays, which Haartman warned against. To build on a rocky island would include cutting down trees and bushes. That would have given the mosquitoes less hiding places and kept them away from the houses. Higher building would also have been an advantage since the domestic mosquitoes fly close to the ground.

Haartman's malaria research consisted of only two theses and in the history of medicine he is mostly remembered for his theoretical work *Sciagraphia morborum*, in which he classified the diseases according to their symptoms [15]. Although malaria continued to be a huge problem in Finland the research would not be continued until the middle of the 19^th ^century. The director of the medical board in Finland, Carl Daniel von Haartman, published a replacement for Haartman's handbook in 1844. He had travelled and studied abroad [29], but his view on malaria cures did not represent anything new or original. Although he prescribed quinine, he also recommended emetics and purgatives [30].

## Conclusions

Two important recommendations changed the treatment of malaria in Sweden during the 18^th ^century. The introduction of Peruvian bark was the first effective drug, but unfortunately it was expensive and remained rare and seldom used. The decision not to bleed malaria patients was essential for the treatment.

Healthcare in the Finnish part of Sweden was primitive and there were only a few physicians. When Johan Haartman was appointed district physician in Turku he travelled widely in the district. To improve the situation he wrote a medical handbook aimed for those who had no opportunity to seek the advice of a phycisian. Haartman recommended Peruvian bark, but he knew that it was out of reach for most people. Therefore, he also recommended other drugs. He also analysed all the commonly used cures and warned against them that he thought were dangerous.

As a scientist Haartman discussed malaria in two thesis. The Southwest parts of Finland had been ravished by severe malaria epidemics in the 1770's. Haartman compared the malaria cases with meteorological data and draw the conclusion that a warm summer correlated with the epidemics next spring.

In another thesis Haartman analysed the malaria situation in archipelago. The number of malaria cases was low in June and July and started to rise in August. Haartman thought that he had found a special kind of malarial fever and called it archipelago fever. Although Haartman did not know the connection between the mosquitoes and the parasite his advice would have been effective. He recommended building the cottages away from the shallow bays in places where the wind could blow. That would have decreased the number of vectors in the cottages. Haartman also wanted to change the daily working hours. He suggested an early rise with a long nap during the day.

Both as a district physician and as a professor Haartman worked diligently to improve general healthcare and medical education. He spread his medical handbook to the parishes by donations and made certain that it became available also in the most remote parts of the country. The Royal Academy of Turku was small and Haartman was the only professor in medicine. His donations provided the means for another medical chair to the university.

## Competing interests

The authors declare that they have no competing interests.

## Authors' contributions

Lena Hulden is the single author of the manuscript.
